# Mammary inflammatory gene expression was associated with reproductive stage and regulated by docosahexenoic acid: in vitro and in vivo studies

**DOI:** 10.1186/s12944-016-0386-1

**Published:** 2016-12-09

**Authors:** Sen Lin, Yalin Zhang, Yanrong Long, Haifeng Wan, Lianqiang Che, Yan Lin, Shengyu Xu, Bin Feng, Jian Li, De Wu, Zhengfeng Fang

**Affiliations:** Key Laboratory for Animal Disease Resistance Nutrition of the Ministry of Education, Animal Nutrition Institute, Sichuan Agricultural University, Chengdu, 611130 People’s Republic of China

**Keywords:** Sow, Mastitis, Periparturient period, Mammary epithelial cells, DHA

## Abstract

**Background:**

Periparturient mastitis is the most prevalent disease affecting lactating animals. However, it has long been relied on antibiotics to deal with mastitis, leading to a potential threat to food safety. This study was aimed to investigate the expression of pro-inflammatory cytokines in mammary glands of sows around parturition when mastitis and oxidative stress usually occur, and evaluate the anti-inflammatory effect of docosahexenoic acid (DHA) in porcine mammary epithelial cells (PMEC) challenged by lipopolysaccharide (LPS).

**Methods:**

Mammary tissues and blood samples were collected from seven pregnant sows at different reproductive stages. Primarily cultured PMEC at passage 4 were assigned to four treatments: basal medium (control), basal medium with LPS (10 μg/mL) (LPS treatment), basal medium with LPS (10 μg/mL) and DHA (100 or 200 μM) (LPS + DHA treatments), and cell samples were harvested after 24 h incubation. The measurements included oxidative stress markers in blood samples and gene expression in mammary tissues and PMEC samples.

**Results:**

Serum α-tocopherol concentration was lower at parturition than at day 90 of gestation and day 28 post parturition, while serum malondialdehyde concentration was higher at day 28 post parturition than at day 90 of gestation. Higher interleukin (IL)-1β mRNA abundance while lower LPS binding protein mRNA abundance in mammary tissues were observed at day 90 of gestation compared with that at parturition and at day 28 and 35 post parturition. Mammary tumor necrosis factor (TNF)-α mRNA abundance were lower at parturition than at day 90 of gestation and day 28 and 35 post parturition, whereas mammary IL-8 mRNA abundance were lower at parturition than at day 35 post parturition. In the PMEC experiment, compared with the control, increased mRNA abundances of Toll-like receptor (TLR)-4 downstream target, myeloid differentiation factor 88 (MyD88), IL-6 and IL-8 were observed in LPS treatment, whereas DHA appeared to decrease mRNA abundances of MyD88, IL-6 and IL-8 induced by LPS.

**Conclusions:**

The down-regulated expression of pro-inflammatory cytokines in mammary tissues and aggravated systemic oxidative stress at parturition suggest that sows are in a vulnerable status during periparturient period. DHA appears to attenuate inflammatory responses in LPS-challenged PMEC through modulation of TLR4 signalling pathway.

## Background

Mastitis is the most prevalent disease affecting lactating animals worldwide [1]. Previous studies demonstrated that prevalence of mastitis in sows was about 13% [2] and it was about 25.5% in lactating heifers in Dutch [3]. For swine, acute clinical mastitis was reported to be associated with reduced piglet growth and survival [4]. It is noteworthy that the prevalence and severity of mastitis is associated with the periparturient period in both humans and dairy cows [5]. Similarly, mastitis for sows is commonly categorized under the term periparturient hypogalactia, which implies the high incidence of mastitis during the periparturient period [4].

It is well recognized that bacteria are the main cause of mastitis. For example, gram-negative coliform bacteria most frequently cause an acute inflammation while gram-positive bacteria like *Staphylococcus aureus* and *Streptococcus uberis* may cause persistent and chronic infection [6]. Nevertheless, why mastitis is most prevalent during periparturient period is still poorly understood. Immune suppression is a natural physiological process that occurs approximately from 1 week prepartum to 1 week postpartum and has been proposed to be related to the high incidence of mastitis during periparturient period [7]. Inflammatory gene expression and systemic oxidative stress status are two crucial factors impacting the immune function of the system, and figuring out how they changed across gestation and postpartum period may strengthen our understanding about the immunological status and high incidence of mastitis during this period. Therefore, the present study was firstly designed to determine the variation rule of inflammatory gene expression and oxidative stress status in sows during periparturient period.

In practice, antimicrobial therapy is still the principle approach to controlling mastitis [8]. However, overuse of antibiotics will cause a series of detrimental results such as development of drug resistant bacteria. Therefore, alternative methods such as nutritional regulation need to be developed to deal with mastitis. We have previously confirmed that consumption of diet rich in n-3 polyunsaturated fatty acids (PUFA) could attenuate mammary inflammation in rats [9]. It has been elucidated that Docosahexaenoic acid (DHA) and Eicosapentaenoic acid (EPA) are capable of attenuating inflammatory responses by antagonizing the Toll-Like-Receptor4 (TLR4) signaling pathway [10, 11]. However, whether n-3 PUFA can also be used to prevent mastitis in sows has not been reported. Moreover, relative trials in sows require mammary tissue sampling which would be costly. Therefore, primarily cultured porcine mammary epithelial cells (PMEC) are used as an in vitro model to test the anti-inflammatory effect of n-3 PUFA in PMEC challenged by lipopolysaccharide (LPS).

## Methods

### Animals and sampling

Use of animals in the current study was approved by the animal care and use committee of Sichuan Agricultural University. A total of seven pregnant sows were included in the study. All sows were fed the same diet and had free access to water during the whole experimental period. Before biopsy, mammary glands were washed, disinfected with ethanol and anesthetized locally with lidocaine. Mammary tissue samples were then collected with a BARD MAGNUM biopsy instrument according to the method of Zhu et al. [12] at day 90 of gestation, at parturition as well as day 28 and 35 post parturition. The samples were immediately frozen in liquid nitrogen and stored at −80 °C until analysis. Blood samples were collected at day 90 of gestation, parturition and day 28 after parturition, standing for 15 min, and supernatants from centrifuged (1500 g, 15 min) samples were stored at −20 °C until analysis.

### Primary culture of PMEC and treatments

Primary culture of PMEC was according to the methods of Zheng et al. [13]. Briefly, porcine mammary gland tissues were obtained from a non-lactating gilt after slaughter. Then a piece of sample approximately 1 cm^3^ was minced into pieces with surgical scissors and the pieces were placed in a 10 cm culture dish. Before incubated in a 37 °C humidified atmosphere with 5%CO_2_, the culture dish was added with 10 mL culture medium. The culture medium was composed of DMEM/F12, 10% fetal bovine serum, 5 μg/mL hydrocortisone, 10 μg/mL insulin, 10 ng/mL epithelial growth factor, 100 μg/mL penicillin and 100 μg/mL streptomycin. After growing to monolayer, the cells were digested by trypsin. When fibroblast cells were separated from the bottom of the dish, most epithelial cells were still attached to the bottom. The digestion was ceased at this time and fibroblast cells were moved to another dish. Afterwards, mammary epithelial cells were digested with trypsin and seeded onto two dishes. After several passages, most cells were mammary epithelial cells. At passage 4, mammary epithelial cells were seeded onto 6-well plates. Till 80% confluence, the cells were assigned to four treatments for 24 h: (1) basal medium (control); (2) basal medium with 10 μg/mL LPS (LPS); (3) basal medium with 10 μg/mL LPS and 100 μM DHA (LPS + 100μMDHA); (4) basal medium with 10 μg/mL LPS and 200 μM DHA (LPS + 200μMDHA).

### RNA extraction and real-time PCR

The mRNA abundances were measured by real-time polymerase chain reaction (PCR). Total RNA from mammary gland tissue or mammary epithelial cells was extracted using a TRIZOL Reagent kit (Invitrogen, Carlsbad, CA). The cDNA was synthesized using a reverse transcription (RT) kit (TAKARA, Japan) following the manufacture’s instructions. Primers (Table 1) were derived from Gene bank and synthesized by a commercial company (Invitrogen, Chengdu, China). Quantitative real-time RT-PCR analysis was carried out using a CFX96 Real-Time PCR Detection System (Bio-Rad, Hercules, CA, USA) with commercial SYBR Green kits (TaKaRa). The specificity of PCR products was examined with melting curve analysis. Relative mRNA expression was calculated with 2^−ΔΔCt^ method using GAPDH as the internal control where ΔΔCt = (Ct target gene unknown sample − Ct GAPDH unknown sample) − (Ct target gene calibrator sample − Ct GAPDH calibrator sample).

**Table 1:** Sequences for real-time PCR primers

Gene		Primer sequences (5′-3′)	Product size	Genebank Accession
IL-1β	Forward	TGACCTGTTCTTTGAGGCTGAC	113 bp	M98820.1
	Reverse	CGAGATGCTGCTGTGAGATTTG		
TNF-α	Forward	CCACTCTGACCCCTTTACTCTGA	154 bp	NM_013693.2
	Reverse	CTGTCCCAGCATCTTGTGTTTC		
IL-8	Forward	CCAGCAGGAAACCAGAAGAAAG	123 bp	NM_001173399.2
	Reverse	CAACTTTGTCACGACCATACCC		
IL-6	Forward	TGAACTCCCTCTCCACAAGC	74 bp	NM_001252429.1
	Reverse	GGCAGTAGCCATCACCAGA		
LBP	Forward	GGCTTCCTTGCTCCTGTCAT	177 bp	NM_001128435.1
	Reverse	GTTGGTGGTCAGTCGGATGT		
TLR4	Forward	TCAGTTCTCACCTTCCTCCTG	166 bp	XM_013986843.1
	Reverse	GTTCATTCCTCACCCAGTCTTC		
MYD88	Forward	GATGGTAGCGGTTGTCTCTGAT	148 bp	NM_001099923.1
	Reverse	GATGCTGGGGAACTCTTTCTTC		
αS1-casein	Forward	ACAAATGAGGACAAGCATACCC	175 bp	NM_001004029.2
	Reverse	GAGGGATGTTGGTGAATAATGG		
GAPDH	Forward	CTACAGCAACAGGGTGGTGGA	179 bp	NM_001206359.1
	Reverse	GGATGGAAACTGGAAGTCAGG		

### Serum concentration of α-tocopherol

Serum concentration of α-tocopherol was detected using a commercial kit (Nanjing Jiancheng bioengineering Institute) according to the manufacture’s instructions. At the beginning of the experiment, blank tube, standard tube and determining tube were designated. Then, 400 μL of double-distilled water and 600 μL of absolute ethanol were added into blank tube. Afterwards, 300 μL of double-distilled water, 100 μL of standard and 600 μL of absolute ethanol were added into standard tube while 300 μL of double-distilled water, 100 μL of serum sample and 600 μL of absolute ethanol were added into determining tube. The mixture was vortexed and 1.2 mL of heptanes were added to each tube. The tubes were vortexed for 1 min and then centrifuged at 3000 rpm for 10 min. 100 μL of reagent 1 and 50 μL of reagent 2 were added to each tube and set for 5 min, after which 50 μL of reagent 3 and 1 mL of absolute ethanol were added. After 2 min’ stand, absorbance of each tube was determined at 533 nm with a spectrophotometer. Then the concentration of α-tocopherol was calculated.

### Serum concentration of malondialdehyde (MDA)

Serum concentration of MDA was determined using a commercial kit (Nanjing Jiancheng bioengineering Institute) according to the manufacture’s instructions. At the beginning of the experiment, blank tube, standard tube,determining tube and control tube were designated. At first, 100 μL of reagent 1 was added to each tube and then 100 μL of absolute ethanol, 100 μL of standard and 100 μL of sample were added to blank tube, standard tube as well as determining tube and control tube, respectively. After mixing the solutions, reagent 2 was added to each tube and then 1 mL of reagent 3 was added to blank tube, standard tube and determining tube while 1 mL of 50% glacial acetic acid was added to control tube. Then the concentration of MDA was calculated.

### Statistic analysis

All statistical analysis was performed with the General Linear Model procedures of SAS statistical package (V8.1, SAS Institute Inc., Cary, NC, USA). Least-squares means comparison was used to evaluate differences among treatments. A value of *P* < 0.05 is used for determination of significant differences.

## Results

### Expression of inflammation related genes in mammary glands of sows during periparturient period

**Fig. 1:**
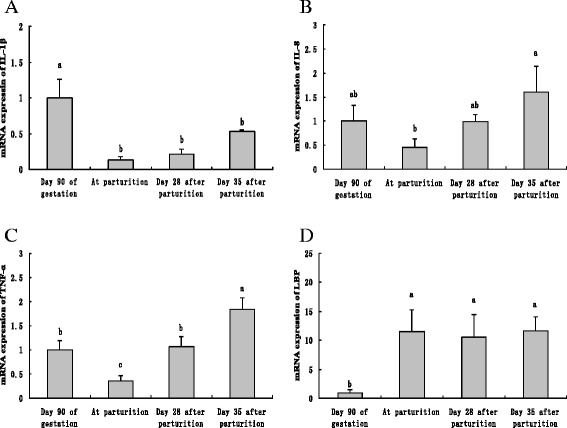
The mRNA abundances of inflammation related genes in sow mammary glands at different reproductive stages. The mRNA abundances of IL-1β (**a**), IL-8(**b**), TNF-α(**c**) and LBP(**d**) were determined by RT-PCR with mammary tissue obtained at day 90 of gestation, parturition as well as day 28 and 35 after parturition. Values are presented as means ± SE. Statistics with no common letters differ significantly (*P* < 0.05)

As shown in Fig. 1, the mRNA abundance of interleukin (IL)-1β at parturition, day 28 and 35 post parturition was lower (*P* < 0.05) than that at day 90 of gestation (Fig. 1a). In contrast, the mRNA abundance of IL-8 at parturition was significantly lower (*P* < 0.05) than that at day 35 post parturition, but was not different from that at day 90 of gestation and day 28 post parturition (Fig. 1b). In addition, the mRNA abundance of TNF-α at parturition was significantly lower (*P* < 0.05) than that at day 90 of gestation and day 28 and 35 post parturition (Fig. 1c). Moreover, the mRNA abundance of TNF-α at day 35 post parturition was also higher (*P* < 0.05) than that at day 90 of gestation and day 28 post parturition. Taken together, the mRNA abundance of inflammatory cytokines in mammary glands tended to decrease from late gestation to parturition, while tended to increase from parturition to lactation. In contrast, the mRNA abundance of LPS binding protein (LBP) was lower (*P* < 0.05) at day 90 of gestation than at parturition and day 28 and 35 post parturition (Fig. 1d).

### Oxidative stress status of sows during periparturient period

**Fig. 2:**
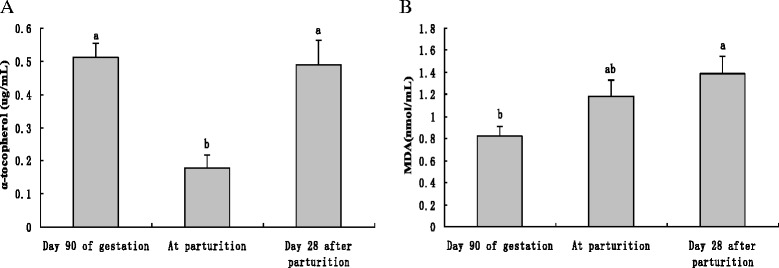
Concentration of α-tocopherol and MDA in serum of sows at different reproductive stages. Serum Concentration of α-tocopherol(**a**) and MDA(**b**) was determined with commercial kits with serum samples obtained at day 90 of gestation, parturition and day 28 after parturition. Values are presented as means ± SE. Statistics with no common letters differ significantly (*P* < 0.05)

The oxidative stress status of sows was also evaluated and presented in Fig. 2. Serum α-tocopherol concentration was lower (*P* < 0.05) at parturition than at day 90 of gestation and day 28 post parturition (Fig. 2a). Serum MDA concentration was higher (*P* < 0.05) at day 28 post parturition than at day 90 of gestation (Fig. 2b).

### Effect of DHA on LPS-stimulated inflammatory responses in PMEC

**Fig. 3:**
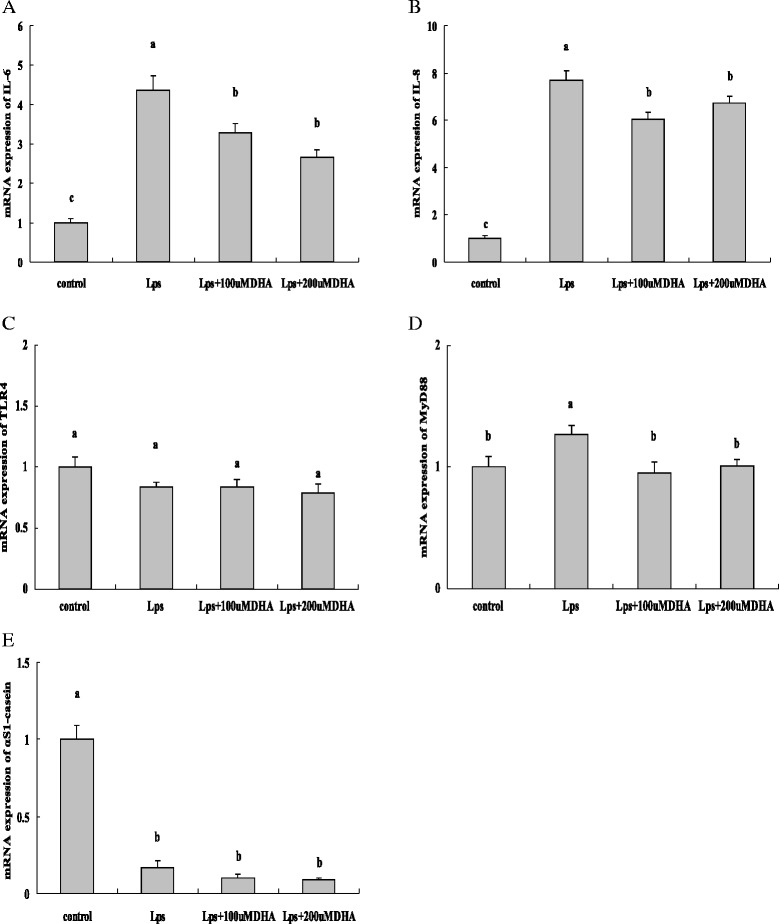
Effect of DHA on gene expression in LPS-stimulated procine mammary epithelial cells. The cells were treated with 10 μg/mL LPS in the absence or presence of DHA (100,200 μM) for 24 h. The mRNA abundances of IL-6(**a**), IL-8(**b**), TLR4(**c**), MyD88(**d**) and αS1-casein (**e**) were determined by RT-PCR. Values are presented as means ± SE. Statistics with no common letters differ significantly (*P* < 0.05)

Compared with the control cultured by the basal medium, the mRNA abundances of IL-6 (Fig. 3a) and IL-8 (Fig. 3b) were significantly increased (*P* < 0.05) in the LPS treatment, indicating the establishment of inflammatory responses. However, the mRNA abundances of IL-6 and IL-8 were lower (*P* < 0.05) in the LPS + DHA treatments than in the LPS treatment. The mRNA abundance of Toll-like receptor (TLR)-4 was not different (*P* > 0.05) among the four treatments (Fig. 3c). However, compared with the control, increased (*P* < 0.05) mRNA abundance of TLR4 downstream target, myeloid differentiation primary response gene 88 (MYD88), was observed in the LPS treatment rather than in the LPS + DHA treatments (Fig. 3d). In addition, compared with the control, decreased (*P* < 0.05) mRNA abundance of αS1-casein was observed in the LPS and LPS + DHA treatments (Fig. 3e).

## Discussion

Despite improvement in management, nutrition and vaccination strategy, mastitis remains a problem for mammals [7]. To date, the mechanisms for the prevalence of mastitis during periparturient period has not been well elucidated. Inflammatory genes are known to play crucial roles in the inflammatory responses. Thus, tracking how they changed during the peripaturient period may be helpful for understanding the peripaturient mastitis. However, information is scarce about the variation rule of inflammatory genes in mammary tissues during periparturient period. Furthermore, oxidative stress, which has been tightly linked to increased production of acute phase cytokines and inflammatory responses [14], is another crucial factor impacting the occurrence of inflammation. Therefore, the first endpoint of this study was to determine the mRNA expression of inflammatory genes in mammary tissues and oxidative stress status of sows during periparturient period.

An important finding in the present study was that the expression of inflammatory cytokines in mammary glands tended to decrease from late gestation to parturition, while tended to increase from parturition to lactation. These results are consistent with a previous study in dairy cows which showed that the trends for IL-6 and IL-8 mRNA abundances in mammary tissues were to decline through involution and increase slightly during early lactation [15]. The dramatic decrease of pro-inflammatory cytokines at parturition may indicate impaired immune function of immune cells in mammary tissues at this time point. Macrophage is the main cell type in healthy mammary tissues and can facilitate neutrophil recruitment by producing interleukin and TNF-α [15]. Moreover, neutrophil can also release TNF-α [16]. Therefore, decreased pro-inflammatory cytokine expression suggests that the immune function of macrophage and neutrophil may decrease with the approach of parturition. A previous study, in which the phagocytic activity of neutrophil and monocyte in blood of sows around parturition was detected, found that the phagocytic activity of both cell types was lowest at parturition [17]. It was also demonstrated that the decrease in neutrophil activity is associated with a higher incidence of mastitis during the period following parturition [18]. In conclusion, the decrease of pro-inflammatory cytokines at parturition can be attributed to the periparturient immunosuppression of mammals. It was elucidated that mammals would go through an immunosuppression process during periparturient period which may be helpful for the survival of offspring by decreasing the immunological rejection to fetus and passing immune cells to fetus [19]. In consequence, around parturition, the immune cells in mammary glands have impaired immune function which may lead to increased susceptibility of mammary tissues to exogenous pathogens and thus contribute to the high incidence of mastitis during this period.

Another finding in this study was that the mRNA abundance of LBP increased rapidly at parturition and remained high until day 35 after parturition. LBP is capable of forming complex by binding to LPS and then binds to CD 14 and stimulates TLR4 signaling pathway [20]. Therefore, following parturition, up-regulated LBP may also increase the susceptibility of mammary glands to exogenous pathogens. In addition, it was discovered that α-tocopherol decreased at parturition and MDA tended to increase following parturition. These results were in agreement with a previous study [21]. α-tocopherol in tissues can control oxidative stress by capturing free radicals and other reactive substances [22] while MDA is a biomarker frequently used to evaluate lipid peroxidation [23]. These results indicate that the anti-oxidative capacity will decrease with the approach of parturition. It was previously reported that LPS stimulation facilitated the recruitment of TLR4 into lipid raft [24], which is ROS dependent [25]. Therefore, the oxidative stress of sows around parturition may also facilitate TLR4 activation by exogenous pathogens and partly contribute to the high prevalence of peripartum mastitis.

Taken together, it was proposed that around parturition, impaired immune function manifested by the decreased pro-inflammatory cytokine mRNA abundance, up-regulated expression of LBP and exaggerated oxidative stress would finally facilitate the invasion of pathogens. Once invaded by pathogen, the inflammatory responses in mammary tissues would be activated, and more pro-inflammatory cytokines would be produced. Thus, to prevent mammary tissues from damage by hyper-inflammation, it may be important to down-regulate the production of pro-inflammatory cytokines at the periparturient period. DHA, a kind of n-3 PUFA, has been proved to exert potential anti-inflammatory effect [26]. We have previously reported that consumption of diet rich in n-3 PUFA could attenuate inflammatory responses in mammary glands of rats [9]. However, few studies have focused on the effect of n-3 PUFA on sow mastitis. To evaluate the effect of DHA on LPS-stimulated inflammatory responses in porcine mammary epithelial cells might provide some references on the potential application of n-3 PUFA in sows. Consequently, the second endpoint of this study was to test the anti-inflammatory effect of DHA using an in vitro mammary inflammation model induced by LPS.

It was observed that mRNA abundances of IL-6 and IL-8 increased significantly following LPS stimulation, indicating the initiation of inflammatory responses in porcine mammary epithelial cells. This result is in consistence with a previous study demonstrating that LPS significantly stimulated the inflammatory genes in bovine mammary epithelial cells [27]. Notably, when DHA was supplemented to the medium, the mRNA abundances of IL-6 and IL-8 decreased significantly, indicating its anti-inflammatory effect in porcine mammary epithelial cells. Additionally, LPS and DHA appeared to have no effect on TLR4 mRNA expression, whereas mRNA expression of MyD88, a crucial downstream modulator of TLR4 in the TLR4 pathway [28], was up-regulated by LPS and down-regulated by DHA. It thereby appeared that LPS did not affect the mRNA expression of TLR4 but increased the TLR4 activation. DHA might attenuate the inflammatory responses by inhibiting the activation of TLR4, suppressing the down stream signaling of TLR4 and thus reducing the production of pro-inflammatory cytokines. αS1-casein is an important composition of porcine milk proteins [29]. Interestingly, in the present study DHA did not show an attenuation effect on LPS-induced inhibition of αS1-casein expression. This might be attributable to the assumption that upon pathogen invasion cells have the first priority to keep them from damage by attenuating pathogen-induced inflammatory responses which might occur before having injured cells restore anabolic functions like milk protein synthesis.

## Conclusions

Taken together, the down-regulated mRNA expression of pro-inflammatory cytokines in mammary tissues and aggravated systemic oxidative stress at parturition suggest that sows are in a vulnerable status which may make it easier for exogenous pathogens to invade the organism and cause inflammatory responses during periparturient period. Moreover, DHA appeared to attenuate LPS-induced inflammatory responses in mammary epithelial cells through modulating the downstream target of TLR4 pathway. These findings have important implication for preventing periparturrient mastitis through dietary inclusion of n-3 PUFA.

## Abbreviations

DHA: Docosahexenoic acid
IL: Interlukin
LBP: LPS binding protein
LPS: Lipopolysaccharide
MDA: Malondialdehyde
MYD88: Myeloid differentiation factor
TLR: Toll-like receptor
TNF: Tumor necrosis factor
